# Changes in insight throughout the natural four-year course of obsessive-compulsive disorder and its association with OCD severity and quality of life

**DOI:** 10.3389/fpsyt.2023.1231293

**Published:** 2023-10-13

**Authors:** Nadja Wolf, Johanna A. M. du Mortier, Patricia van Oppen, Adriaan W. Hoogendoorn, Anton J. L. M. van Balkom, Henny A. D. Visser

**Affiliations:** ^1^Mental Health Care Institute Geestelijke gezondheidszorg (GGZ) Centraal, Amersfoort, Netherlands; ^2^Department of Psychiatry, Amsterdam Public Health, Amsterdam University Medical Center, Vrije Universiteit, Amsterdam, Netherlands; ^3^Geestelijke gezondheidszorg (GGZ) inGeest, Specialized Mental Health Care, Amsterdam, Netherlands

**Keywords:** insight, OCD, obsessive-compulsive disorder, natural course, severity, quality of life

## Abstract

**Objective:**

Patients with obsessive-compulsive disorder (OCD) and poor insight show higher symptom severity, lower quality of life (QoL), and a reduced treatment response compared to patients with good insight. Little is known about changes in insight. This study explored the course of insight and its association with OCD severity and QoL among 253 patients with OCD participating in the prospective naturalistic Netherlands Obsessive Compulsive Disorder Association (NOCDA) Study.

**Results:**

In 70% of the participants with available insight data, the level of insight changed during the four-year course. Insight was most variable in participants with poor insight. Improvement of insight scores was statistically significantly associated with improvement of Y-BOCS scores (*r* = 0.19), but not with changes in QoL scores. Change in insight in the first 2 years was not statistically significantly predictive of OCD severity or QoL at four-year follow-up.

**Conclusion:**

These findings suggest that patients’ levels of insight may change during the natural four-year course of OCD and that improvement in the level of insight have a positive association with improvement in OCD severity.

## 1. Introduction

The concept of insight in obsessive-compulsive disorder (OCD) has gained growing attention. In clinical terms, insight is the extent to which a patient recognizes that OCD thoughts and behaviors are excessive or unreasonable (1). Traditionally, patients with OCD have been described as having good insight into their symptoms. However, it is now recognized that patients with OCD vary in the degree of insight regarding the accuracy of the beliefs underlying their obsessive-compulsive symptoms. Therefore, the DSM-5 has included a specifier for the level of insight, which may be classified in “good and fair insight” (“the individual recognizes that OCD beliefs are definitely or probably not true or that they may or may not be true”), “poor insight” (“the individual thinks the OCD beliefs are probably true”) and “absent insight” (“the individual is completely convinced that OCD beliefs are true”). These levels of insight occur at every level of OCD severity (2).

Poor insight occurs in about 15 to 36% of patients with OCD (3–9), and has been associated with worse clinical characteristics in terms of greater severity of OCD symptoms (3–5, 8–11), higher comorbidity rates (2, 7, 9, 10, 12), longer duration of illness (7, 8), lower quality of life (QoL) (8), and more chronicity (versus episodic OCD) (2, 13). Visser et al. (2) demonstrated that poor insight predicted a more unfavorable two-year natural course concerning OCD severity compared to patients with good or fair insight. Further, several studies have suggested that patients with poor insight showed a reduced treatment response when treated with psychotropic medication, (4, 5, 7) Cognitive Behavior Therapy (CBT) (14–18), or deep brain stimulation (19). Poor insight is associated with neuropsychological differences compared to patients with good insight, such as lower empathic concern and a biased emotion recognition (20) and impairments in response inhibition, verbal memory and fluency (21). It is also associated with neurobiological differences such as lower levels of brain-derived neurotrophic factor in plasma (22) and abnormal small-world brain functional networks in patients with OCD and poor insight (23).

While several studies have investigated the association between the level of insight and several aspects of OCD, only a few have investigated changes in insight. Research has shown that insight can improve after treatment with CBT (18, 24–26), with pharmacological treatment (3, 4, 6) or with combined treatment (8). In addition, it has been shown that treatment can lead to a shift from poor insight to fair or even good insight with concomitant reduction in OCD symptom severity (4, 8). Thus, insight seems to be a dynamic phenomenon rather than a static one.

The extent to which changes in insight contribute to the natural course of OCD severity has not been established. Recent literature emphasizes the relevance of using both OCD severity and QoL as markers for treatment response (27). However, it is understudied how changes in insight, OCD severity and QoL are related to each other. The aim of the present study is to gain further knowledge about the course of insight and its association with the natural course of OCD severity and QoL. If improvement in insight has a positive association with the natural course of OCD severity or QoL, and improvement in insight remains stable over time, addressing insight in OCD treatment specifically might be beneficial. Given the relatively small sample size of the study population, this is an explorative study. The goals of this study are to: (i) describe the course of insight, (ii) investigate the association between changes in insight and changes in OCD severity and changes in QoL, and (iii) investigate the association between change in insight and the four-year course of OCD severity and QoL.

## 2. Materials and methods

### 2.1. Design and participants

Data were obtained from the Netherlands Obsessive Compulsive Disorder Association (NOCDA) study, a multicenter naturalistic cohort study designed to investigate the naturalistic long-term course of OCD in patients referred to a mental health care center for evaluation and treatment. A detailed description of the rationale, objectives and methods of NOCDA is described elsewhere (28). Recruitment took place from 2005 to 2009, at seven Dutch second-line mental health care centers. All patients diagnosed with OCD and referred to one of the participating mental health care centers were asked permission to be contacted for research purposes during the intake procedure. All patients who consented were contacted and invited to participate in the study, irrespective of the stage of the disorder, the OCD subtype, the presence of co-morbidity and the stage of chronicity. A total of 419 adults (aged 18 years and over) with a lifetime diagnosis of OCD, as determined by the administration of the Structured Clinical Interview for DSM-IV Axis I Disorders (SCID-I) (29) prior to recruitment to the study, were included. No formal exclusion criteria were applied except for an inadequate understanding of the Dutch language. Comprehensive measurements were performed at baseline and after 2, 4 and 6 years at one of the participating mental health care centers by a trained and experienced research nurse or psychologist. During the follow-up, participants received treatment as usual that was based on the Dutch multidisciplinary guidelines. The study protocol was approved by the local ethical committee and written informed consent was obtained from all participants (28).

In the NOCDA study, an insight measure was assessed at first 2 years after the original baseline measure, which is the baseline of the present study. For the purpose of the current study, only participants with OCD complaints at our baseline and for whom data were available on insight in OCD were included (*N* = 253). There were no significant baseline differences regarding gender, age, comorbidity, Y-BOCS severity and QoL within the included NOCDA sample and the participants included in this study.

### 2.2. Measurements

#### 2.2.1. Insight

The Overvalued Ideas Scale (OVIS) was used to assess insight into OCD symptoms. The OVIS is designed to assess the severity of overvalued ideas in OCD in the previous week (30). The concept of overvalued ideas can be considered equivalent to the specifiers poor or absent insight as described in DSM-5. The OVIS is a clinician-administered 10-item scale that reflects: strength, reasonableness and accuracy of belief; the extent to which others share beliefs; attribution of similar or differing views; the effectiveness of compulsions; the extent to which the disorder has caused the belief; and the strength of the resistance to the belief. Each item of the OVIS is rated from 0 to 10, with higher scores indicating poorer insight. The scores are summed and divided by 10 to create a total score. Internal consistency (*α* = 0.88–0.95) and inter-rater reliability (*r* = 0.86) are adequate.

Good insight was indicated by an OVIS score of ≤3.9, fair insight was reflected by an OVIS score of ≥4 and ≤ 5.9, poor insight was indicated by an OVIS score of ≥6 and ≤ 7.4 OR ≥ 6 plus a score lower than 9 for the accuracy item (the belief is somewhere between totally inaccurate and almost accurate), and absent insight was reflected by an OVIS score ≥ 7.5 plus a score of 9 or higher for the accuracy item (the belief is nearly or completely accurate). Due to small group sizes, the latter two groups were combined for the statistical analyzes. We treated insight as a categorical variable (i.e., good, fair and poor insight), since these cut-off scores were previously employed in the adult OCD literature (2, 31, 32), and because previous research suggested that only poor insight has a negative impact on the course of OCD (2). The OVIS was assessed at our baseline, 2 years after baseline, and 4 years after baseline. Data were present in 253 (100%), 181 (72%) and 170 (67%) participants, respectively. Complete OVIS data at baseline, and 2 and 4 years after baseline, were available in 142 participants.

#### 2.2.2. OCD severity

The clinician-rated Yale-Brown Obsessive-Compulsive Scale (Y-BOCS) was used to assess OCD severity (33). This is a 10-item scale assessing the severity of obsessions and compulsions in the previous week, with total scores ranging from 0 (no symptoms) to 40 (extreme symptoms). The Y-BOCS severity scale has well-documented validity and reliability (33). The Y-BOCS was assessed at our baseline, 2 years after baseline, and 4 years after baseline. Data were present in, respectively, 253 (100%), 220 (87%) and 205 (81%) participants. Complete Y-BOCS data at baseline, and 2 and 4 years after baseline, were available in 194 participants.

#### 2.2.3. Quality of life (QoL)

The self-rated EuroQol five-dimensional questionnaire (EQ-5D) was used to assess the QoL. This instrument has demonstrated its suitability and reliability in the general population and is also applicable in patient samples (34). The EQ-5D contains five dimensions significant for QoL: mobility, self-care, daily activities, pain/discomfort, and depression/anxiety. Each dimension is rated at three levels: no problems, some problems, and major problems. These health states are converted into an index score – the EQ-5D – which represents the generic overall QoL. The EQ-5D index score ranges between 0 (indicating the worst possible health) and 1 (reflecting the best possible health). The EuroQol was assessed at our baseline, 2 years after baseline, and 4 years after baseline. Data ware present in 222 (88%), 203 (80%), and 184 (73%) participants, respectively. Complete EuroQol data at baseline, and 2 and 4 years after baseline, were available in 163 participants.

#### 2.2.4. OCD severity and QoL

To establish *OCD and other DSM-IV-TR Axis I disorders*, the Structured Clinical Interview for DSM-IV-TR (SCID-I) (29) was administered. To assess the number of current comorbid mental disorders, the ascertained diagnoses on the SCID-I were counted. Severity of *depressive and anxiety symptoms* were assessed using the Beck Depression Inventory (35) and the Beck Anxiety Inventory (36). To ensure the quality of the data, all assessors received training and supervision in performing the measurements. Further, all interviews where audiotaped and monitored by randomly checking about 10% of all taped interviews.

### 2.3. Statistical analyzes

Means, standard deviations and percentages were calculated to summarize demographics and clinical measures. A Pearson’s correlation coefficient was calculated to examine the correlation between insight and OCD severity, insight and QoL, and OCD severity and QoL. Clinical characteristics of participants with and without missing OVIS data at two- or four-year follow-up were compared using *t*-tests for continuous variables and chi-square statistics for categorical variables.

To describe the four-year course of insight, a descriptive analysis was conducted using frequencies and percentages. We first determined whether the level of insight changed or remained stable during the four-year course. A changed level of insight was defined as either an improvement or a deterioration in level of insight (i.e., poor, fair or good insight) in the first 2 years or the last 2 years. An unchanged level of insight was defined as a stable level of insight during the four-year course, e.g., poor insight at baseline, two- and four-year follow-up. Next, in order to establish whether changes in insight can be maintained over time, we noted where the level of insight of participants with poor, fair and good insight at baseline subsequently stood at two-year follow-up and at four-year follow-up.

To investigate the association between the changes in insight and changes in OCD severity across several occasions, we estimated repeated measure correlations for determining the common within-individual association between insight and OCD (37, 38). Similarly, we investigated the association between changes in insight and changes in quality of life (total EQ-5D-score).

A linear regression analysis was used to determine whether change in insight (i.e., OVIS score) in the first 2  years predicted the four-year course of OCD severity (i.e., Y-BOCS score) adjusted for baseline insight and baseline OCD severity. Similarly, we investigated the relationship between changes in insight in the first 2 years and its association with the four-year course of quality of life (i.e., EQ-5D score).

Statistical analyzes were performed using Statistical Package for Social Sciences (SPSS, version 27).

## 3. Results

### 3.1. Sample description

At baseline, the mean age of the sample was 39.1 years (SD = 10.8). The sample consisted of 143 women (56.5%) and 110 men. Of the sample, 76% had current OCD. Table 1 shows the sociodemographic and clinical characteristics of the sample. Participants had a mean score of 16.8 (SD = 8.1) on the Y-BOCS, reflecting a moderate mean severity of OCD. Approximately 20% of our sample consisted of participants with a severe Y-BOCS score of 24 or higher. Baseline level of overvalued ideation as measured by the OVIS was 4.3 (SD = 1.5, range: 1.0–8.2), reflecting a fair mean level of insight. The mean EQ-5D utility score was 0.75 (SD = 0.25), indicating a poor QoL (39). At least one DSM-IV comorbid psychiatric disorder was diagnosed in 30% of participants.

**Table 1:** Sociodemographic and clinical characteristics of the study sample.

Variable	M (SD)
*Baseline*	
Age (years)	39.1 ± 10.8
Female, no (%)	143 (56.5)
Paid job (% yes)	59.0
Living with a partner (% yes)	55.6
No. of DSM-IV Axis I disorders current	1.3 ± 1.1
Affective disorder (% yes)	15.8
Anxiety disorder besides OCD (% yes)	21.3
Other Axis 1 disorder (% yes)	8.7
Severity depressive symptoms (BDI score)	12.0 ± 10.2
Severity anxiety symptoms (BAI score)	14.1 ± 11.4
Age at onset of OCD	17.7 ± 9.2
OCD symptom severity:	
Y-BOCS obsessions	8.3 ± 4.3
Y-BOCS compulsions	8.5 ± 4.5
Y-BOCS total score	16.8 ± 8.1
Insight: OVIS	4.3 ± 1.5
Quality of Life: EuroQoL-5D	0.75 ± 0.25
*Two-year follow-up*	
OCD symptom severity: Y-BOCS total score	16.4 ± 8.9
Insight: OVIS	4.4 ± 1.3
Quality of Life: EuroQoL-5D	0.72 ± 0.26
Contact with a psychologist or psychiatrist (% yes)	66.0
Use of antidepressant or antipsychotic medication (% yes)	41.9
*Four-year follow-up*	
OCD symptom severity: Y-BOCS total score	16.2 ± 9.4
Insight: OVIS	4.5 ± 1.3
Quality of Life: EuroQoL-5D	0.74 ± 0.26
Contact with a psychologist or psychiatrist (% yes)	63.8
Use of antidepressant or antipsychotic medication (% yes)	38.7

OCD, Obsessive-Compulsive Disorder; Y-BOCS, Yale-Brown Obsessive-Compulsive Scale; OVIS, Overvalued Ideas Scale; BDI, Beck Depression Inventory; BAI, Beck Anxiety Inventory; SD, standard deviation.

A comparison was made on clinical characteristics between participants with and without missing data on insight at two- or four-year follow-up. No significant differences were found.

A Pearson’s correlation showed a moderate positive correlation between the OVIS and Y-BOCS at baseline (*r* = 0.45, *p* < 0.001), two-year follow-up (*r* = 0.40, *p* < 0.001), and four-year follow-up (*r* = 0.39, *p* < 0.001). The OVIS and EuroQol correlated weakly to moderately negative at baseline (*r* = −0.18, *p* = 0.007), two-year follow-up (*r* = −0.26, *p* < 0.001), and four-year follow-up (*r* = −0.31, *p* < 0.001).

Between baseline and two-year follow-up, 66% of the participants had contact with a psychologist, psychotherapist or a psychiatrist; the mean (SD) number of contacts was 18 (34.0); 15% had >7 days of day time or clinical treatment; 41.9% used antidepressant or antipsychotic medication at two-year follow up. Between two- and four-year follow-up, 63.8% of the participants had contact with a psychologist, psychotherapist or a psychiatrist; the mean (SD) number of contacts was 16 (27.3); 14% had >7 days of day time or clinical treatment; 38.7% used antidepressant or antipsychotic medication at four-year follow-up.

### 3.2. The four-year course of insight

**Figure 1:**
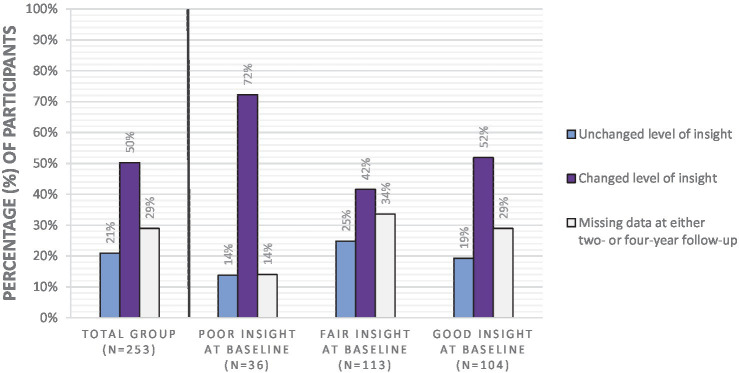
Percentage of participants with unchanged and changed levels of insight during the four-year course of insight.

**Figure 2:**
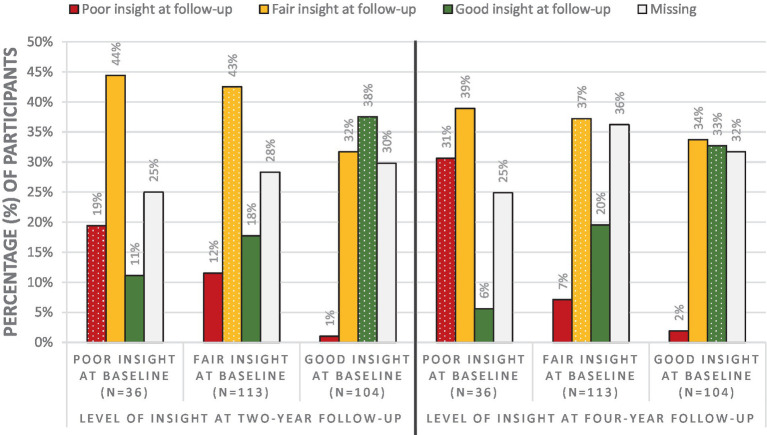
Percentage of participants with poor, fair and good insight at two- and four-year follow-up subdivided per insight level at baseline. The dotted columns indicate that there was no change in level of insight.

We started by examining whether the level of insight changed or remained stable during the four-year course, both for the total group of participants and for the three levels of insight: poor, fair or good insight at baseline, using descriptive statistics (see Figure 1). Next, in order to see if changes in insight can be maintained over time, we noted where the level of insight of participants with poor, fair and good insight at baseline subsequently stood at two-year follow-up and at four-year follow-up (see Figure 2). Figures 1, 2 show the data including participants with missing data. Below, we will describe the four-year course of insight of the participants with available OVIS-data.

In the total group of participants with available OVIS-data, in 30% of the participants the level of insight remained stable during the four-year course, while changes in level of insight occurred in 70% of the participants.

When focusing on participants with poor insight at baseline and available OVIS-data, the majority of the participants (84%) showed changes in level of insight during the four-year course. In 74% of the group with poor insight at baseline, the level of insight improved to fair or good insight in the first 2 years. At four-year follow-up, 59% of the participants with poor insight at baseline no longer belonged to the poor insight group.

Looking at the group with fair insight at baseline and available OVIS-data, the level of insight remained fair in 37% of participants, while in 63% the insight level either improved to good or deteriorated to poor somewhere during the four-year course. In most cases, participants with fair insight at baseline still had fair insight at four-year follow-up. When these individuals do shift from insight level, they more often shift to good insight than to poor insight.

When focusing on the group with good insight at baseline and available OVIS-data, the level of insight remained good in 27% of the participants during the four-year course, while 73% of this group showed changes in their level of insight. At four-year follow-up, 3% of the participants had poor insight, 49% had fair insight, and 48% had good insight. Thus, participants with good insight at baseline almost never end up with poor insight at four-year follow-up.

Taken together, these results suggest that the level of insight changes more often than it remains stable during a four-year course. Of the three insight groups, insight was most variable in participants with poor insight. The group with poor insight at baseline can shift to good insight, and it is less likely for people with good insight to shift to poor insight.

### 3.3. The association between changes in insight and changes in OCD severity and quality of life

The results of the repeated measures correlation investigating the association between changes in insight and changes in OCD severity showed that change in insight was significantly associated with change in OCD severity [*r_rm_* (S.E.) = 0.19 (0.04), *p <* 0.001, 95%-CI = (0.12, 0.27)]. This result is equivalent to a Cohen’s *d* of 0.40 and indicates a small to medium positive relationship, where participants whose insight improved tended to have an improvement in OCD severity.

The results of the repeated measures correlation investigating the association between changes in insight and changes in QoL showed no association between change in insight and change in quality of life [*r_rm_* (S.E.) = −0.03 (0.04), *p =* 0.47, 95%-CI = (−0.12, 0.05)].

### 3.4. The association between change in insight and the four-year course of OCD severity

Results of the linear regression analysis showed that change in insight in the first 2 years did not statistically significantly affect the four-year course of OCD severity with small-to-medium effect size when controlling for baseline level of OCD severity and baseline level of insight (beta = 0.147, *p* = 0.067, see Table 2). Similarly, changes in insight in the first 2 years did not statistically significantly affect the four-year course of QoL with negligible effect size when controlling for baseline level of QoL and baseline level of insight (beta = 0.031, *p* = 0.734, see lower part Table 2).

**Table 2:** Parameter estimates of linear regression analyzes with four-year OCD severity (upper part) and quality of life (lower part) as outcome variable estimating the effect of change in insight in the first 2 years when adjusted for baseline outcome and for baseline insight.

Variables	*b*	95%-CI	beta	*p*-value
*Outcome variable: 4-year Y-BOCS*				
Change (2 vs. 0 years) in OVIS	0.92	(−0.07, 1.92)	0.147	0.067
Baseline OVIS	0.63	(−0.43, 1.69)	0.104	0.240
Baseline Y-BOCS	0.63	(0.48, 0.78)	0.629	<0.001
*Outcome variable: 4-year EQ-5D*				
Change (2 vs. 0 years) in OVIS	0.01	(−0.03, 0.04)	0.031	0.734
Baseline OVIS	−0.03	(−0.07, 0.00)	−0.166	0.08
Baseline EQ-5D	0.54	(0.36, 0.71)	0.468	<0.001

OCD, Obsessive-Compulsive Disorder; OVIS, Overvalued Ideas Scale; Y-BOCS, Yale-Brown Obsessive-Compulsive Scale; EQ-5D, EuroQol five-dimensional questionnaire; b, unstandardized regression coefficient; CI, confidence interval; beta, standardized regression coefficient.

## 4. Discussion

The aims of the present study were to describe the four-year course of insight, investigate the association between changes in insight and changes in OCD severity and QoL, and to investigate the association between change in insight and the four-year course of OCD severity and QoL.

In describing the natural four-year course of insight, we found that the level of insight changed more often than it remained stable. Of participants with available insight data, changes occurred in 70% of cases, while the level of insight remained stable in 30% of the group. Insight was most variable in participants with poor insight. The majority with poor insight showed changes in level of insight, 59% with available OVIS data of whom no longer exhibited poor insight after 4 years. This finding is consistent with that of Catapano et al. (4), who found that 46% of patients with poor insight shifted to good insight at the end of a three-year observational period after pharmacological treatment. Similarly, Matsunagu et al. (8) found that the level of insight of 56% of the group with poor insight improved to fair or good insight 6 months after combined cognitive behavioral treatment and medication.

We found that changes in insight were correlated with changes in OCD severity with a small to medium effect size, indicating that improvement in insight and OCD severity are associated. This finding is congruent with previous research that reported a positive correlation between change in insight and change in OCD severity (3, 4, 6). We found no association between changes in insight and changes in QoL.

Finally, we found that change in insight in the first 2 years was not statistically significantly predictive of OCD severity or QoL after 4 years. A possible explanation for this might be the time frame of 2 years between each time point. This time frame might be too long to test whether changes in insight predict OCD severity and QoL. Due to small group sizes, we were not able to test whether maintaining or acquiring poor insight (OVIS >6) has a negative impact on the course of OCD severity. Future research should address this, since previous research indicated that only poor insight had a negative impact on the course of OCD, whereas no differences were found with regard to the course of OCD between patients with good insight and those with fair insight (2). In addition, previous research suggested that when using OVIS as a categorical variable, patients with poor insight showed altered brain activation during a symptom provocation task compared to patients with good/fair insight, whereas a continuous approach of the OVIS showed less evidence for this association (40). Therefore, it might be that there is a threshold beyond which worse insight matters for the course of OCD, for altered brain activation and maybe also for worse response to treatment.

Strengths of this study include the large sample of participants with OCD who were followed for a long period of time and the use of a valid and reliable measure of insight in OCD. Several limitations also have to be addressed. First, there was a considerable amount of missing data at follow-up in this study. This may have biased our results. However, no significant differences were found for baseline data between participants with and without missing data. Second, our sample consisted of individuals with a mild to moderate OCD severity, as some of the participants in our study no longer had current OCD. Consequently, the heterogeneity of our sample was large, making our research applicable to an OCD population of patients who suffered or still suffer from OCD. However, the results may not be generalized to the entire population of individuals with OCD. A third limitation is related to the establishment of the OVIS cut-off scores for categorizing good, fair and poor insight. Although these cut-off scores have been used in previous studies, their validity still remains to be established. A fourth limitation, as already mentioned, is the time frame of 2 years between each time point. Future research should include multiple assessment points with smaller time frames in order to establish the directionality of changes in insight and OCD severity and QoL. Despite these limitations, this study is the first study, to our knowledge, to explore the course of insight and its association with OCD severity and QoL. Further research with larger samples are needed to expand on our findings. A direction of special interest is the neuropsychological and neurobiological differences in patients with poor insight. It is interesting to establish whether changes in insight are also related to neuropsychological and neurobiological changes such as changes in executive functions and brain-derived neurotrophic factors in plasma.

Taken together, our findings suggest that a patient’s level of insight can change, and that improvement in insight might be beneficial for the course of OCD severity. These findings of this study have clinical implications. First, assessment of insight in OCD during treatment may be important, as it might be a factor that needs specific attention. Further, our findings underscore the importance of aiming to enhance insight during treatment (18).

## Data availability statement

The original contributions presented in the study are included in the article/supplementary material, further inquiries can be directed to the corresponding author.

## Ethics statement

The studies involving humans were approved by Medical Ethical Committee VUmc. The studies were conducted in accordance with the local legislation and institutional requirements. The participants provided their written informed consent to participate in this study.

## Author contributions

NW, JM, PO, AH, AB, and HV contributed to the conception of the present study. NW and AH performed the statistical analyzes. NW took the lead in writing the manuscript. All authors contributed to the article and approved the submitted version.
